# Study of the effect of altitude on the measurement of glycated haemoglobin using point-of-care instruments

**DOI:** 10.5830/CVJA-2014-078

**Published:** 2015

**Authors:** Sandra W Veigne, Eugene Sobngwi, Brice E Nouthe, Eric V Balti, Serge Limen, Mesmin Y Dehayem, Vicky Ama, Jean-Louis Nguewa, Jean-Claude Mbanya, Joelle Sobngwi-Tambekou, Maimouna Ndour-Mbaye, Alioune Camara, Naby M Balde

**Affiliations:** National Obesity Center, Yaounde Central Hospital and Faculty of Medicine and Biomedical Sciences, University of Yaounde 1, Yaounde, Cameroon; National Obesity Center, Yaounde Central Hospital and Faculty of Medicine and Biomedical Sciences, University of Yaounde 1, Yaounde, Cameroon; Molecular Medicine and Metabolism Laboratories, Bio-technology Center, University of Yaounde 1, Yaounde, Cameroon; National Obesity Center, Yaounde Central Hospital and Faculty of Medicine and Biomedical Sciences, University of Yaounde 1, Yaounde, Cameroon; Department of Medicine, McGill University, Montreal, Quebec, Canada; National Obesity Center, Yaounde Central Hospital and Faculty of Medicine and Biomedical Sciences, University of Yaounde 1, Yaounde, Cameroon; Diabetes Research Center, Faculty of Medicine and Pharmacy, Brussels Free University-VUB, Brussels, Belgium; National Obesity Center, Yaounde Central Hospital and Faculty of Medicine and Biomedical Sciences, University of Yaounde 1, Yaounde, Cameroon; National Obesity Center, Yaounde Central Hospital and Faculty of Medicine and Biomedical Sciences, University of Yaounde 1, Yaounde, Cameroon; National Obesity Center, Yaounde Central Hospital and Faculty of Medicine and Biomedical Sciences, University of Yaounde 1, Yaounde, Cameroon; National Obesity Center, Yaounde Central Hospital and Faculty of Medicine and Biomedical Sciences, University of Yaounde 1, Yaounde, Cameroon; National Obesity Center, Yaounde Central Hospital and Faculty of Medicine and Biomedical Sciences, University of Yaounde 1, Yaounde, Cameroon; Molecular Medicine and Metabolism Laboratories, Bio-technology Center, University of Yaounde 1, Yaounde, Cameroon; University of Technology, Kingston, Jamaica; Centre of Higher Education in Health Sciences, Catholic University of Central Africa, Yaounde, Cameroon; Cheick Anta Diop University, Dakar, Senegal; University Teaching Hospital of Donka, Conakry, Guinea; University Teaching Hospital of Donka, Conakry, Guinea

**Keywords:** glycated haemoglobin, altitude, diabetes

## Abstract

We measured the glycated haemoglobin (HbA_1c_) levels of a total of 24 non-diabetic volunteers and diabetic patients using a point-of-care (POC) analyser in three Cameroonian cities at different altitudes. Although 12 to 25% of duplicates had more than 0.5% (8 mmol/mol) difference across the sites, HbA_1c_ values correlated significantly (*r* = 0.89–0.96). Further calibration studies against gold-standard measures are warranted.

## Abstract

HbA_1c_ concentration is used for the appropriate diagnosis and management of diabetes,^1^,^2^ but the standard way of measurement requires an expensive and time-consuming ion-exchange, high-performance liquid chromatography (HPLC) technology. Point-of-care (POC) instruments represent a cheaper alternative to determine HbA_1c_ levels in five to 10 minutes. They can be used by non-laboratory staff to tailor a patient’s care and educational messages to HbA_1c_ values and clinical findings in a one-stop-shop approach.^3^,^4^ Their potential shortcomings include cases of haemoglobinopathy or some environmentally linked limitations.^5^,^6^

While operating temperature and humidity are easily controlled, altitude cannot be standardised for operation. We investigated the performance of one of the most commonly used POC HbA_1c_ instruments in African clinical settings, situated at varying altitudes.

## Methods

In this cross-sectional study, HbA_1c_ concentrations were measured in three cities of Cameroon in blood samples simultaneously collected from the same individuals. The study settings were Douala (13-m altitude), Yaounde (650-m altitude), and Bamenda (1 600-m altitude).

The study was approved by the National Ethics Committee of Cameroon. All participants gave their informed consent.

The study participants were 24 volunteers distributed in four groups: six non-diabetic (healthy) volunteers [no clinical symptoms, fasting glycaemia < 1.26 g/dl (6.99 mmol/l) and HbA_1c_ levels < 6.6% (< 49 mmol/mol)], six patients with diabetes with HbA_1c_ levels < 6.6% (< 49 mmol/mol), six patients with HbA_1c_ levels at 6.6–8.0% (49–64 mmol/mol) and six patients with HbA_1c_ levels > 8.0% (> 64 mmol/mol).

All patients had to have had diabetes for at least one year, with stable treatment and HbA1c values over at least three months preceding the study defined by HbA_1c_ variation < 1% between two measurements. Exclusion criteria included any haemoglobinopathy, recent malaria, haematological disorder or any other acute medical condition in the preceding month, total haemoglobin level > 11 g/dl, and creatinine clearance < 60 ml/min.

Volunteers were invited, and after informed consent, we conducted an interview, clinical examination and biochemical investigations for the ascertainment of eligibility. Collections of venous blood in eligible participants were all done the same day from an antecubital vein in four EDTA tubes stored in refrigerated containers for all three assays.

The blood samples collected on the same day for each participant were immediately transported by car to the target settings in a refrigerated container. The room temperature was standardised for all study sites at 25°C, and humidity was maintained between 45 and 60%.

HbA_1c_ measurements were performed using the In2it POC device (Bio-Rad laboratories, Deeside, UK), which was calibrated prior to the study, with all reagents from the same lot (072T128). The same operator performed the assays in each of the settings within 48 hours of blood collection. All manipulations were done following the operating procedure of the manufacturer in order to reduce the variability of the measurements.

## Statistical analysis

Using SPSS 17.0, data were analysed and expressed as mean ± standard deviation. Comparisons across the groups were done using analysis of variance, and associations were verified by Spearman’s correlation. Agreement between methods was assessed using Bland and Altman plots of the difference against the means of the two methods.

## Results

Participants were 12 males and 12 females, aged 54 ± 15 years. Their mean body mass index was 28.9 ± 5.8 kg/m^2^, mean systolic and diastolic blood pressures were 128 ± 18 and 77 ± 8 mmHg, respectively, and mean haemoglobin was 13.4 ± 1.8 g/dl. The duration of diabetes in all patients was 10 ± 6 years with a pre-inclusion HbA1c value of 7.8 ± 2.3%.

Overall, there was no statistically significant difference between mean HbA1c measurements across the sites (Table 1). The correlation between measurements varied from *r* = 0.89, *p* < 0.001 between the 650-m/1 600-m altitudes, *r* = 0.92, *p* < 0.001 between the 13-m/650-m altitudes, to *r* = 0.96, *p* < 0.001 between 13-m/1 600-m altitudes. The coefficient of variation (CV) was 3.4% for the 650-m/13-m duplicates, 5.1% for 1 600-m/13-m duplicates and 3.2% for 1 600-m/650-m duplicates.

**Table 1:** Comparison of mean HbA_1c_ levels by group across the sites

	*Point-of-care In2it analyser*			
*Study group*	*Douala (13 m)*	*Yaounde (650 m)*	*Bamenda (1 600 m)*	*p-value*
Healthy controls	5.0 ± 0.6	5.4 ± 0.3	5.6 ± 0.5	0.15
Patients with diabetes				
HbA_1c_ < 6.5% (< 49 mmol/mol)	5.9 ± 0.6	5.7 ± 0.6	5.9 ± 0.4	0.29
HbA_1c_ > 8.0% (> 64 mmol/mol)	8.1 ± 3.0	7.9 ± 3.1	8.0 ± 3.0	0.66
All study participants	8.4 ± 1.8	8.5 ± 1.7	9.0 ± 2.2	0.84
All study participants	6.8 ± 2.2	6.9 ± 2.2	7.1 ± 2.3	0.31

**Fig. 1.**
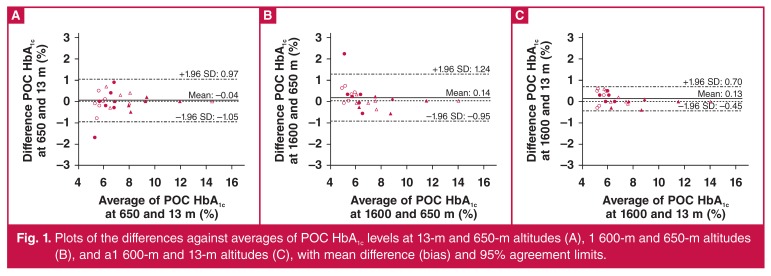
Plots of the differences against averages of POC HbA1c levels at 13-m and 650-m altitudes (A), 1 600-m and 650-m altitudes (B), and a1 600-m and 13-m altitudes (C), with mean difference (bias) and 95% agreement limits.

The mean differences expressed as estimates (95% CI) in percentages between measurements at two different sites were –0.04 (–1.05−0.97%), +0.14 (0.95−1.24%) and +0.13 (–0.45−0.70%), respectively, between the 650-m/13-m (Fig. 1A), 1 600-m/650-m (Fig. 1B), and 1 600-m/13-m altitudes (Fig. 1C).

The HbA_1c_ differences were > 0.5% (8 mmol/mol) in 3/24 (12%) between the 1 600-m/13-m measurements, 4/24 (17%) between the 650-m/13-m measurements and in 6/24 (25%) between the 1 600-m/650-m measurements. In only one case associated with more than one percentage difference across sites was a patient with one of the readings at 4.2% (22 mmol/mol) in one site, which normally would have prompted a second check. We did not find any differences in the percentage variation of HbA_1c_ levels at the low (*n* = 12), medium (*n* = 6) and high (*n* = 6) values for the different study sites, namely 650-m/13-m (*p* = 0.453), 1 600-m/650-m (*p* = 0.111) and 1 600-m/13-m altitudes (*p* = 0.344).

## Discussion

This study indicates that the POC analyser showed no significant differences across Cameroonian sites located at altitudes varying from 13 to 1 600 m (≤ 0.5% in 75% of comparisons). Although measurements were not repeated in each site to reflect clinical practice, our results suggest a test reliability of the In2it POC instrument below 1 600 m.

Interestingly, previous studies in which the device calibration was performed with HPLC, had suggested satisfactory external validity.^7^ This was however not investigated in our study and therefore represents a major limitation with the sample size.

However, considering our findings and the cut-off value of 3.5% of CV for optimal performance between laboratories (between study sites in our case), one could say that although no significant difference was observed between HbA1c levels at the three altitudes, the POC apparatus had a relatively high variability between 13 and 1 600 m.^8^ As expected, this variability was higher in low and normal HbA1c levels (not shown).

In this regard, the use of the POC HbA_1c_ analyser could be more indicated for the monitoring of patients with a view to comparing before- and after-treatment glucose control, especially in the lower values, even in the absence of calibration with an HPLC machine.

Consistent with our results, a recent study of HbA1c variations in Chinese populations living at different altitudes did not find meaningful variations in the HbA1c levels and the estimated average glucose levels of patients living in different sites.^9^

However, on the one hand, Ju *et al.*^9^ in their study used the immunoturbidimetric method for the measurement of HbA_1c_ levels (also without validation against the gold standard for HbA_1c_ measurement), while we used a baronate affinity chromatography to separate glycated from non-glycated haemoglobin for photometry.^4^,^9^ On the other hand, we sought to evaluate the possible effect of altitude on the accuracy of a POC HbA_1c_ analyser in patients with diabetes, while they aimed to evaluate whether altitude could modify the glycation of HbA_1c_.

In our study, we observed that 12–25% of duplicates had more than a 0.5% (8 mmol/mol) difference across the sites. The performance of POC apparatus in general and the In2it in particular has (independent of altitude) been assessed before. These investigations constituted a body of evidence showing the need for improvement in the performance of devices for optimal care.^10^-^12^

The recent performance of these devices has given promising results. This also was the case where the In2it apparatus is concerned, despite the between-batch variability of results, which still needs to be addressed.^7^,^13^ To circumvent this in our study, we used reagents from the same lot number at all study sites. However, in daily clinical practice, this could indeed be a concern for patients’ follow up.

With the generalisation of HbA1c use, especially in developing countries that have limited access to an HPLC and have a wide variety of physical environments, it is important to know which parameters should be taken into account when validating POC HbA_1c_ devices, which are commonly presented as the adequate alternative to estimate glycaemic control of patients.

## Conclusion

Our results reinforce the need for calibration of POC instruments against the HPLC in each setting used, to ensure validity of the readings. We did not find any significant differences when measuring HbA_1c_ levels at different altitudes on the same samples. However this requires validation with further studies, using larger sample sizes and addressing situations with higher proportions of patients with haematological disorders.
